# Methicillin-Resistant *Staphylococcus aureus* (MRSA) Strain ST398 Is Present in Midwestern U.S. Swine and Swine Workers

**DOI:** 10.1371/journal.pone.0004258

**Published:** 2009-01-23

**Authors:** Tara C. Smith, Michael J. Male, Abby L. Harper, Jennifer S. Kroeger, Gregory P. Tinkler, Erin D. Moritz, Ana W. Capuano, Loreen A. Herwaldt, Daniel J. Diekema

**Affiliations:** 1 Department of Epidemiology, University of Iowa College of Public Health, Iowa City, Iowa, United States of America; 2 Center for Emerging Infectious Diseases, University of Iowa College of Public Health, Iowa City, Iowa, United States of America; 3 Department of Medicine, University of Iowa Carver College of Medicine, Iowa City, Iowa, United States of America; 4 Program of Hospital Epidemiology, University of Iowa Hospitals and Clinics, Iowa City, Iowa, United States of America; 5 Department of Pathology, University of Iowa Carver College of Medicine, Iowa City, Iowa, United States of America; University of Würzburg, Germany

## Abstract

**Background:**

Recent research has demonstrated that many swine and swine farmers in the Netherlands and Canada are colonized with MRSA. However, no studies to date have investigated carriage of MRSA among swine and swine farmers in the United States (U.S.).

**Methods:**

We sampled the nares of 299 swine and 20 workers from two different production systems in Iowa and Illinois, comprising approximately 87,000 live animals. MRSA isolates were typed by pulsed field gel electrophoresis (PFGE) using *Sma*I and *Eag*I restriction enzymes, and by multi locus sequence typing (MLST). PCR was used to determine SCC*mec* type and presence of the *pvl* gene.

**Results:**

In this pilot study, overall MRSA prevalence in swine was 49% (147/299) and 45% (9/20) in workers. The prevalence of MRSA carriage among production system A's swine varied by age, ranging from 36% (11/30) in adult swine to 100% (60/60) of animals aged 9 and 12 weeks. The prevalence among production system A's workers was 64% (9/14). MRSA was not isolated from production system B's swine or workers. Isolates examined were not typeable by PFGE when *Sma*I was used, but digestion with *Eag*I revealed that the isolates were clonal and were not related to common human types in Iowa (USA100, USA300, and USA400). MLST documented that the isolates were ST398.

**Conclusions:**

These results show that colonization of swine by MRSA was very common on one swine production system in the midwestern U.S., suggesting that agricultural animals could become an important reservoir for this bacterium. MRSA strain ST398 was the only strain documented on this farm. Further studies are examining carriage rates on additional farms.

## Introduction

*Staphylococcus aureus* is one of the most common and devastating human pathogens [1]. Though approximately a third of the population is colonized with *S. aureus* [2], [3], colonization by strains of *S. aureus* that are resistant to methicillin (methicillin-resistant *S. aureus*, MRSA) is less common. A recent publication estimated that 1.5% of the United States (U.S.) population (∼4.1 million persons) is colonized with MRSA [4]. Klevens et al. recently showed that deaths from MRSA infections in the U.S. have eclipsed those from many other infectious diseases, including HIV/AIDS. On the basis of data from several major metropolitan areas in the U.S., these investigators estimated that MRSA caused 94,000 infections and over 18,000 deaths in the U.S. in 2005 [5].

Moreover, MRSA has been found in a variety of animals, including horses [6], [7], cattle [8], dogs, cats [9], and swine [10], [11], [12]. Voss et al. reported that the prevalence of MRSA among pig farmers was >760 times higher than that among patients admitted to Dutch hospitals [13]. Multi locus sequence typing (MLST) suggested that these MRSA isolates belonged to sequence type 398 (ST398), and had been transmitted from pigs to pig farmers, among pig farmers and their family members, and from the colonized son of a swine veterinarian to a hospital nurse. A subsequent study found that 4.6% of veterinarians and veterinary students were colonized with MRSA compared with a population-based estimate of 1% [14].

Additional studies in swine have shown that isolates obtained from swine and their human caretakers are frequently indistinguishable, suggesting transmission between the two animal species [11], [12]. Indeed, investigations in the Netherlands demonstrated that ST398 now accounts for 20% of all MRSA detected in that country, documenting the importance of considering livestock and other animals when examining the epidemiology of MRSA [15]. However, despite research examining swine-associated MRSA in the Netherlands and Canada [10], [12], currently the prevalence of MRSA in swine or their caretakers is unknown in the U.S. In a rural state such as Iowa, which produces 25% of the swine raised in the U.S., transmission of MRSA on swine farms or in veterinary facilities could complicate efforts to reduce MRSA transmission statewide and beyond. Therefore, we conducted a pilot culture survey to examine the prevalence of MRSA in swine and swine workers in two swine farming production systems in Iowa and Illinois.

## Materials and Methods

### Description of farms and swine sampled

Production system A (PSA) is a conventional commercial confinement operation consisting of a 5200 head breed-to-wean sow farm with multiple age-segregated nurseries, finishing, and wean-to-finish sites scattered throughout northern Illinois and eastern Iowa. Collectively, approximately 60,000 swine are present at any one time. Sows in this herd originated from both Canada (Manitoba, Ontario and Quebec) and the U.S. (Minnesota and Illinois). The crossbred sows are from a major swine genetic supplier. The sow herd is relatively young, having been repopulated in 2006. Samples (n = 210) were taken from swine housed at 7 geographically distinct farms within this closed system. The nares of adult sows and swine ages 9, 12, 15, 18, 21, and 24 weeks were swabbed (30 per age group). Animals were not co-mingled prior to sampling. One sample was eliminated because it became contaminated. Nine weeks after the first visit, 20 additional cultures were obtained from swine that were initially 12 and 15 weeks old (10 from each age group). Sixteen weeks after the first visit, 20 samples were obtained from randomly selected dam-piglet pairs.

Production system B (PSB) is also a relatively young herd sow herd comprising approximately 2600 sows at the single sow farm location and 27,000 total animals housed at multiple, age-segregated nursery, finisher and wean-to-finish sites throughout eastern Iowa. The sow farm was populated in 2006 with crossbred females originating solely in the United States (Michigan). The breeding stock females in this herd are also from a major swine genetic supplier, but different than those of PSA. Thirty samples were taken from swine in each of 3 age groups: adult sows, and pigs at 11 and 20 weeks of age (n = 90). Animals were not co-mingled prior to sampling.

### Human participants

Human caretakers (n = 20) provided nasal and oropharyngeal swabs. Employees filled out a questionnaire providing demographic data, potential risk factors for MRSA infection, information about contact with swine, and use of personal protective equipment. The institutional review board (IRB) and the institutional animal care and use committee (IACUC) approved the protocols. All human participants gave written informed consent prior to enrollment.

### Sample collection and bacterial isolation

One naris from each animal and both nares from caretakers were sampled with sterile swabs. Cultures were done as described previously [12]. Briefly, samples were collected using sterile swabs and inserted into Stuart's medium at 4°C for transportation. Samples were inoculated into 2 mL enrichment broth containing 10 g tryptone/L, 75 g NaCl/L, 10 g mannitol/L and 2.5 g yeast extract/L. After 24 h incubation at 35°C, a loopful of broth was inoculated onto selective MRSA agar plates (BBL CHROMagar MRSA, Becton, Dickinson and Company). These plates were incubated 24–48 hours at 35°C and examined for MRSA. Isolates were confirmed to be *S. aureus* by examining their appearance on Gram stain, and by doing the catalase test, the tube coagulase test and the *S. aureus* latex agglutination assay (Pastorex Staph-plus, Bio-Rad). Methicillin resistance was confirmed by testing for the presence of penicillin binding protein 2 (PBP2′) (MRSA latex agglutination test, Oxoid Ltd., Hants, UK). MRSA isolates were stored at −80°C.

### Molecular testing

All human isolates and 15 isolates from swine (representing all age groups) were selected for molecular typing. Pulsed field gel electrophoresis (PFGE) was performed as previously described [16]. Isolates that were non-typeable after *Sma*I digestion were examined after digestion with *Eag*I. Isolates from this study were compared with the type strains for USA100, USA300, and USA400 [17]. For SCC*mec* typing and *pvl* PCR, genomic DNA was extracted using the Wizard Genomic DNA preparation kit (Promega). The multiplex SCC*mec* PCR included ten primer sets: CIF2 F2/R2, mecI P2/P3, RIF5 F10/R13, dcs F2/R1, mecA P4/P7, kdp F1/R1, SCCmec III J1 F/J1 R, ccrB2 F2/R2, SCCmec V J1 F/J1 R, and ccrC F2/R2 [18]. The presence of *pvl* was determined by an additional PCR [19]. Multi locus sequence typing (MLST) was performed on a subset of isolates which were identical by PFGE and analyzed as previously described [20]. All molecular procedures employed known positive and negative controls.

### Antimicrobial susceptibility testing

All human MRSA isolates and the 15 swine isolates evaluated by molecular typing were tested for antimicrobial susceptibility by the broth dilution method described by the Clinical and Laboratory Standards Institute [21]. Isolates were tested for susceptibility to penicillin, oxacillin, tetracycline, erythromycin, clindamycin, trimethoprim-sulfamethoxazole, quinupristin/dalfopristin, gentamicin, levofloxacin, moxifloxacin, linezolid, daptomycin, vancomycin, and rifampin.

### Survey/data analysis

Questionnaire and laboratory data were linked by a unique specimen number. Initially, potential risk factor associations were assessed with Fisher's exact test. Bivariate and multivariate modeling of risk factors were performed by exact logistic regression. A trend in prevalence of MRSA in swine by age group was tested with the Cochran-Armitage trend test. A significance level of 0.05 was used in the analyses. Analyses were performed using SAS software version 9.1 (SAS Institute Inc., Cary, NC).

## Results

### MRSA prevalence in swine

**Figure 1:**
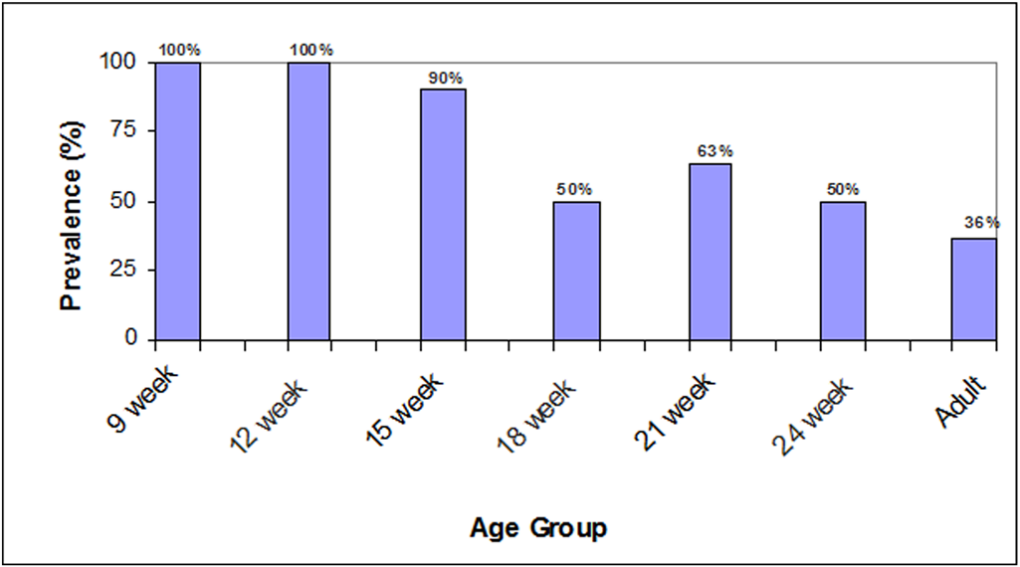
Prevalence of MRSA in swine from production system A by age aroup.

Nasal swabs were taken from 209 swine representing 7 different age groups at PSA. The overall prevalence of MRSA was 70% (147/209). Figure 1 illustrates the significant decreasing trend in prevalence found with the increase in age group (Cochran-Armitage trend test, p-value <0.01). Swine 15 weeks or younger had higher odds of MRSA colonization (OR: 2.17, 95% confidence interval = 1.6 to infinity) when compared to adult swine.

At a follow-up visit 9 weeks later, 20 of 20 samples obtained from the youngest animals were positive. Twelve of 20 (60%) samples obtained 16 weeks after the first visit from 10 randomly selected piglet-dam pairs were positive. Results were concordant for 4 pairs (in 1 pair both dam and piglet had negative nares cultures; in 3 pairs both animals had positive nares cultures) and discordant for 6 pairs (in 2 pairs the dam's nares culture grew MRSA and the piglet's culture did not; in 4 pairs the piglet's nares culture grew MRSA and the dam's culture did not).

Swine from a second production system (PSB) were also tested. Because the prevalence of MRSA carriage was high among younger pigs at PSA, we sampled fewer age groups at PSB. We collected 90 samples from 3 age groups (11 weeks, 20 weeks, and adult). We did not detect MRSA in any of these swabs.

### MRSA prevalence in humans and risk factors for MRSA carriage

Persons working in these swine facilities were invited to participate in the study. PSA employed 18 staff who had contact with swine at the sow farm; 14 (77%) volunteered to provide swabs and respond to a questionnaire. PSB employed 7 staff in contact with swine at the sow farm; 6 (86%) participated in our study. Overall, 9/20 (49%) carried MRSA, all of whom were employed at PSA (9/14 persons sampled at PSA, 64% prevalence). Seven persons were colonized in the nares only and 2 were colonized in both the nares and throat. As all swine and human samples obtained at PSB were negative for MRSA, only PSA was included in the risk factor analyses. Age, gender, use of tobacco products, underlying medical conditions, respiratory illness in the prior 12 months, use of antimicrobial agents in the prior 3 months, exposure to healthcare facilities (including long-term-care facilities), a history of skin and soft tissue infections or of having MRSA in the prior 12 months, duration of employment, the number of swine contacted per day, eating pork products, and exposure to raw pork were not associated with nasal carriage of MRSA (see Table 1). All14 PSA subjects work with breeding swine. Persons who do not obtain blood or other specimens from swine (separate analyses) were at higher risk of carrying MRSA than staff that did do these chores.

**Table 1:** Characteristics of Production System A swine workers and MRSA prevalence.

Variable	Response	N	MRSA positive (%)
Gender			
	Female	1	0 (0.0)
	Male	13	8 (61.5)
Age group			
	<31	6	3 (50.0)
	31–43	3	3 (100)
	> = 44	5	2 (40.0)
Tobacco			
	No	6	4 (66.7)
	Yes	8	4 (50.0)
Lung problems			
	No	12	6 (50.0)
	Yes	2	2 (100)
Heart problems			
	No	13	8 (61.5)
	Yes	1	0 (0.0)
Chronic medical problem			
	No	11	7 (63.6)
	Yes	3	1 (33.3)
Respiratory illness with fever in last 12 months			
	No	10	7 (70.0)
	Yes	4	1 (25.0)
Missed work because of respiratory illness in last 12 months			
	No	10	7 (70.0)
	Yes	4	1 (25.0)
Taken antibiotics in the past 3 months			
	No	11	7 (63.6)
	Yes	3	1 (33.3)
Visited hospital in past 12 months			
	No	9	7 (77.8)
	Yes	5	1 (20.0)
Visited long-term care facility in past 12 months			
	No	11	6 (54.6)
	Yes	3	2 (66.7)
You or family member work in hospital or long-term care facility			
	No	10	7 (70.0)
	Yes	4	1 (25.0)
Diagnosed with skin of soft tissue infection in past 12 months			
	No	14	8 (57.1)
	Yes	0	0 (0.0)
Diagnosed with MRSA in past 12 months			
	No	14	8 (57.1)
	Yes	0	0 (0.0)
Length of employment (years)			
	< = 3	5	4 (80.0)
	4–14	6	3 (50.0)
	> = 14	3	1 (33.3)
Perform cleaning in the swine farm*			
	No	5	5 (100)
	Yes	9	3 (33.3)
Obtain blood or other specimens from swine*			
	No	10	8 (80.0)
	Yes	4	0 (0.0)
Average number of swine you are exposed in a typical day			
	< = 2400	3	2 (66.7)
	2401–5000	8	3 (37.5)
	> = 5000	3	3 (100)
Consume pork products			
	No	1	0 (0.0)
	Yes	13	8 (61.5)
Frequency of consuming pork products			
	2–3 times per week	4	2 (50.0)
	Approximately once per week	6	4 (66.7)
	Less than once per week	2	2 (100)
	More than 4 times per week	1	0 (0.0)
Frequency of handling raw pork products			
	2–3 times per week	3	0 (0.0)
	Approximately once per week	3	3 (100)
	Less than once per week	7	5 (71.4)

* Significant at 95% confidence level using Fisher exact.

### Molecular typing

**Figure 2:**
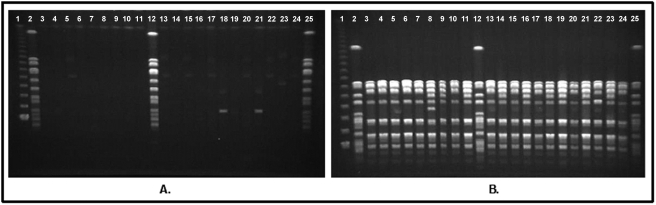
A: PFGE of MRSA isolates from swine and swine workers; DNA digested with *Sma*I. B: PFGE of MRSA isolates from swine and swine workers; DNA digested with *Eag*I. Lane 1: molecular weight ladder. Lanes 2, 12, 25: NCTC 8325 (control strain). Lanes 3–11: isolates from swine workers. Lanes 13–24: isolates from swine.

As previously described [22], the isolates from swine and caretakers were not typeable when the DNA was digested with *Sma*I (Fig. 2a) but were typeable when *Eag*I was used (Fig. 2b). All isolates from swine and from swine workers were closely related by the Tenover criteria [23] and they were distinct from common human strains (USA100, USA300, and USA400, not shown).

All isolates were SCC*mec* type V and *pvl*-negative (data not shown). MLST analysis of a subset of isolates confirmed that these isolates were ST398.

### Antibiotic resistance

All isolates from swine and from humans were resistant to penicillin, oxacillin and tetracycline, and all were susceptible to trimethoprim-sulfamethoxazole, gentamicin, levofloxacin, moxifloxacin, linezolid, daptomycin, vancomycin, and rifampin. Three of 15 (20%) swine isolates were resistant to erythromycin, 2 (13%) were resistant to quinupristin-dalfopristin, and 13 (87%) were resistant to clindamycin. None of the 9 human isolates tested were resistant to erythromycin or quinupristin-dalfopristin, but 1 (11%) was resistant to clindamycin. The unusual pattern of erythromycin susceptibility and clindamycin resistance among 11 of the isolates was confirmed with repeat testing.

## Discussion

This study is the first to document MRSA in U.S. swine and swine workers, and to our knowledge, the first to report the presence of ST398 (also reported as non-typeable MRSA, NT-MRSA) [15] in the U.S. Like previous studies in Canada, Denmark, and the Netherlands [11], [12], [24], ST398 was found in both animals and humans, suggesting transmission between the two. The prevalence of MRSA colonization among swine and swine workers was high at one farm system that we examined in the Midwestern U.S., suggesting that agricultural animals could become an important reservoir for this bacterium. Strain ST398 was the only MRSA identified among the swine and swine workers. This strain has been the predominant strain among swine in the Netherlands and Canada. However, Khanna et al. in Canada recently found both ST398 and ST5/USA100 colonizing the nares of swine and swine workers [12]. This difference may indicate that the epidemiology of MRSA on Canadian swine farms is different than on the affected farm system in Iowa and Illinois. On the other hand, the difference may have resulted from differing sampling methodologies. Khanna et al. sampled a small number of humans and swine on 20 farms whereas we took a larger number of samples from a smaller number of farms in two corporate systems. Furthermore, because we did not type all isolates in this pilot study, additional strain types may be present that we did not detect.

The rate of MRSA colonization in both humans and swine on the farms in one of the corporate systems in our study was high, suggesting that once MRSA is introduced, it may spread broadly among both swine and their caretakers. Other investigators have postulated that this spread may be facilitated by use of tetracycline in swine farming [10]. The ST398 isolates identified in our study were resistant to tetracycline, and thus, could have been selected by antimicrobial pressure on this farm. However, both production systems that we sampled employ similar protocols for prophylactic and therapeutic use of antimicrobial agents, including tetracycline. Therefore, our data do not allow us to speculate on the relationship between antimicrobial use and MRSA carriage. In addition to tetracycline resistance, we found an unusual macrolide-lincosamide resistance phenotype among a subset of isolates (erythromycin susceptible, clindamycin resistant), one which is not explained by the commonly-recognized mechanisms of macrolide-lincosamide resistance [25].

At present, we do not know why one farm system had a high prevalence rate of MRSA among its swine and its swine handlers. The two production systems did have several differences. First, they raised different breeds of swine. Second, PSA was an older, more established operation that had approximately twice the number of animals as PSB. Additionally, a portion of the sows at PSA were imported from Canada, while those from PSB originated in Michigan. Canada is the most important exporter of live hogs to the U.S. [26]. Thus, it is possible that ST398 may have been brought into the U.S. via live swine or pork products. However, this study was not designed to identify the source of the MRSA and additional research should further examine this question.

In addition, our survey did not help us understand why a high proportion of PSA staff carried MRSA. Most of the potential risk factors examined were not statistically different between the carriers and the non-carriers. We cannot explain the observation that staff who do not obtain blood and other samples from the animals were more likely to be carriers than were staff who obtained such samples. Additional studies in larger populations will be needed to identify risk factors and to assess whether this association is real.

Investigators in other countries have documented that ST398 causes infections in humans [11], [13], [15] and Wulf et al. have recently described a hospital-based outbreak in the Netherlands [27]. Iowa ranks first in the nation in swine production, with over 19 million hogs at any time point distributed over more than 10,000 farms [28], [29]. Therefore, one would expect that Iowa would be a good state in which to assess the prevalence of infections caused by ST398 among humans. None of the swine workers in this small study reported prior MRSA infections. In addition, we have not identified this strain among the hundreds of human MRSA isolates examined in several ongoing studies of MRSA (including invasive infections) in Iowa [30], [31].

Our study had several limitations. We demonstrated that MRSA can remain in a population of swine for up to 6 months. However, we did not re-test the same animals over time. Thus, we cannot comment about duration of carriage in particular animals and we could not determine whether the lower rate of colonization in older animals observed at PSA was a true difference related to biological mechanisms or an incidental finding. The latter observation contrasts with prior research that found no significant difference in the rates of MRSA carriage by age group [12]. In addition, we did not evaluate whether the environment was contaminated and could have been a source of transmission for swine or for humans, or whether transmission occurred through direct contact with a colonized animal or human. Moreover, we studied only 9 farms in 2 production systems. Thus, our results may not be generalizable to other swine farms in Iowa and Illinois or to other areas of the U.S.

In summary, we report the first isolation of MRSA from swine and swine workers in the U.S. Although the extent of this problem in the U.S. is currently unknown, our findings may have important implications for the epidemiology of MRSA disease. For example, Van Loo et al. identified MRSA in meat products in the Netherlands [32], suggesting that persons who handle raw pork products might be at risk for acquiring MRSA. Future studies should assess the risk of MRSA disease among swine workers and their contacts, survey retail meat products for MRSA contamination, study larger populations of swine and humans to define the epidemiology of MRSA within swine operations, and assess MRSA carriage rates in other livestock.
